# The impact of physical adjunctive interventions on outcomes of clear aligner treatment: A systematic review of randomized controlled trials

**DOI:** 10.1371/journal.pone.0346566

**Published:** 2026-04-08

**Authors:** Mohamad Radwan Sirri, Mohammad Osama Namera, Mohamad Yaman Salahi Alasbahi

**Affiliations:** Department of Orthodontics, Faculty of Dentistry, Damascus University, Damascus, Syria; Danube Private University, AUSTRIA

## Abstract

**Introduction:**

Physical adjunctive interventions (PAIs), including vibration devices and low-level laser therapy, are promoted to accelerate tooth movement, improve aligner tracking, and reduce discomfort in clear aligner treatment (CAT), but randomized evidence remains inconsistent. This systematic review aimed to assess whether PAIs improve CAT outcomes in terms of objective tooth-movement metrics, aligner tracking, overall treatment efficiency, and patient-centered outcomes, and to appraise the risk of bias and the certainty of the evidence at the outcome level.

**Methods:**

Comprehensive electronic searches of PubMed, Embase, Scopus, Web of Science, and CENTRAL were conducted from database inception (earliest available indexing date in each database) to 30 June 2025 (coverage cut-off). Grey literature searching and hand-searching were also performed, with no restrictions on language or publication status. Eligible studies were human randomized controlled trials (RCTs) comparing CAT combined with PAIs versus CAT alone or sham interventions. Two independent reviewers performed study selection and data extraction, with risk of bias assessed using the Cochrane RoB 2 tool and certainty of evidence appraised with GRADE. Random-effects meta-analyses were conducted when feasible; otherwise, results were narratively summarized. The protocol was prospectively registered (PROSPERO CRD420251132229).

**Results:**

Seven RCTs involving 266 participants were included. No significant improvement in Little’s Irregularity Index (maxilla MD = 0.08, p = 0.869; mandible MD = 0.44, p = 0.487). Vibration improved aligner tracking under a 7-day change schedule (p = 0.003) but not case completion (p = 0.999). Overall compliance was approximately 77%, and no between-group difference was observed (p = 0.390). Pain was slightly lower on days 1–3 only (p < 0.05); no quality-of-life benefits were observed; periodontal indices remained unchanged; biomarkers showed inconsistent results. The certainty of evidence was low to very low, suggesting that further well-designed RCTs are likely to change the effect estimates and may alter the conclusions.

**Conclusions:**

Across objective tooth-movement metrics, aligner tracking, treatment efficiency, and patient-centered outcomes, current randomized evidence does not demonstrate a consistent benefit of physical adjunctive interventions in clear aligner treatment. Interpretation is limited by outcome-level risk of bias concerns and low to very low certainty of evidence.

## Introduction

Clear aligner treatment (CAT) has evolved from a concept introduced in the 1940s to a mature, fully digital modality [1]. Transparent, removable aligners are preferred for their esthetics and convenience and support superior oral hygiene compared with fixed orthodontic appliances, which are associated with greater plaque accumulation, white-spot lesions, and gingival inflammation [2]. In non-extraction orthodontic treatment of mild-to-moderate crowding cases, the median treatment duration is approximately 14–22 months, and is mainly determined by the aligner-change protocol, patient compliance, and the number and extent of refinement stages [3]. Compared with fixed appliances, evidence on overall treatment duration with clear aligners is mixed: some studies report a several-month reduction [4,5], others report no meaningful difference [6,7], and longer treatment times have been reported, particularly in extraction-based protocols [8,9]. The aligner-change interval is pivotal, traditionally around 14 days. Shortening this interval without supportive adjuncts risks loss of tracking and often fails to shorten overall time because refinements increase [10,11]. Selected trials suggest that dedicated acceleration modalities—particularly high-frequency vibration—may permit faster switching with improved tracking and fewer aligners [12].

In response to these challenges, physical adjunctive interventions (PAIs)—non-invasive modalities designed to accelerate orthodontic tooth movement—have been introduced, including mechanical vibration, low-level laser therapy/photobiomodulation (LLLT/PBMT), pulsed electromagnetic fields (PEMF), and low-intensity electrical stimulation (LIES) [13]. Most of these modalities were originally developed for fixed appliances and were subsequently adapted to CAT with the aim of reducing the number of aligners and appointments while maintaining tracking accuracy [14].

These modalities enhance the native mechanobiologic response, primarily by modulating key regulators of bone remodeling [15]. On the pressure side, they promote osteoclast activity through RANKL/OPG pathway modulation [16], while on the tension side, they stimulate osteogenesis via Wnt/β-catenin signaling and sclerostin regulation [17]. This coordinated acceleration of the bone remodeling cycle shortens the initial lag phase, facilitating more efficient tooth movement within biologically safe parameters [13,18].

Preliminary studies indicate that adding PAIs to CAT may reduce overall treatment time [19] and improve tracking [20]. Some reports have also indicated improved patient compliance [21]. For pain outcomes, benefits have been observed at specific intervals [20], whereas other studies found no meaningful effect [21]. Moreover, several investigations detected no additional improvement in oral health–related quality of life [22].

Available systematic reviews evaluating adjunctive physical interventions used with clear aligner therapy are limited and largely modality-specific, with heterogeneous findings and variable certainty, resulting in differing conclusions [12,13,23]. Some have evaluated various acceleration methods used with CAT, without specifically focusing on PAIs [24]. Others have examined the effect of PAIs on orthodontic tooth movement across both fixed appliances and aligners [13]. A smaller subset has focused solely on the effect of vibration on orthodontic tooth movement, often incorporating nonrandomized designs and using suboptimal risk-of-bias tools [25]. Importantly, some reviews apply appropriate risk-of-bias tools only at the study level [13], a practice that Cochrane cautions can mask outcome-specific bias [26]. Collectively, these limitations highlight the need for a comprehensive review that specifically evaluates PAIs in CAT, encompasses all relevant outcomes, and prioritizes human randomized controlled trials (RCTs).

Accordingly, this systematic review aimed to systematically collate and critically appraise RCTs on PAIs in CAT, and to estimate their overall impact and clinical utility. It also applied outcome-level risk-of-bias assessments (RoB 2) and provided rigorous GRADE judgments on the certainty of evidence.

## Materials and methods

### Scoping search

Before initiating this review, a PubMed scoping search was conducted to confirm the absence of prior systematic reviews on the effects of PAIs in CAT; none were identified. The review was prepared in accordance with PRISMA and the Cochrane Handbook.

### Question and eligibility criteria

This systematic review evaluated whether PAIs improve clinical, biologic, and patient-reported outcomes of CAT compared with no adjunct or sham interventions in RCTs.

Eligibility criteria were pre-specified using the PICOS framework (Population, Intervention, Comparison, Outcomes, Study design; see **Table 1**). Excluded studies were: nonrandomized designs (retrospective/observational studies, case series/reports), secondary syntheses (systematic reviews, meta-analyses, narrative reviews), and animal or in-vitro experiments; trials comparing PAIs with other active acceleration methods (e.g., surgical corticotomy, pharmacologic agents, nonphysical adjuncts); and reports with insufficient extractable data (incomplete outcomes or unavailable full text).

**Table 1:** PICOS framework and the searched electronic databases.

Participants	Healthy individuals of any age, sex, or ethnicity undergoing clear aligner therapy, with or without extraction plans; no restrictions on malocclusion type or treatment indication.
Interventions	Nonsurgical physical adjuncts intended to accelerate orthodontic tooth movement (e.g., mechanical vibration devices, low-level light/photobiomodulation [LLLT/LED], bioelectrical stimulation).
Comparisons	Clear aligner treatment without any acceleration adjunct or a sham device/protocol
Outcomes	Total treatment time, Little’s Irregularity Index, PCPDI (reduction), tracking accuracy, pain, oral health–related quality of life, biomarkers, periodontal indices, and root resorption.
Study design	Human randomized controlled trials only, using either parallel-group or split-mouth designs.

LED: Light-Emitting Diode; LLLT: Low-Level Laser Therapy; PCPDI: Proximal Contact Point Discrepancy Index.

### Search strategy

The protocol was registered in PROSPERO on 22 August 2025 (CRD420251132229) before formal screening and data extraction. Database searches were conducted on 25 August 2025 and covered records from inception to 30 June 2025 (coverage cut-off), with no restrictions on language, publication status, or date. Searches were performed in PubMed, Embase, Scopus, Web of Science, and the Cochrane Central Register of Controlled Trials (CENTRAL), complemented by the TRIP database and Google Scholar. Grey literature was searched in OpenAIRE and EBSCO Open Dissertations. Reference lists were hand-searched, and four core orthodontic journals were screened (AJODO, EJO, Journal of Orthodontics, and Orthodontics & Craniofacial Research). Full strategies are provided in S1 Table. Searches and record management were performed by MRS & MYSA, whereas title/abstract screening was performed independently by MRS & MON according to prespecified criteria.

### Study selection and data extraction

Titles and abstracts were screened independently by two reviewers (MRS, MON) against prespecified criteria. Disagreements were resolved by third-reviewer adjudication (MYSA). Eligible records underwent full-text assessment with reasons for exclusion documented. Data were extracted by one reviewer and independently verified in full by a second reviewer using a standardized, pilot-tested form. The form captured: (1) study identifiers and design (e.g., author/year, country/setting, trial design, sample size per arm); (2) participant characteristics and baseline orthodontic status; (3) CAT protocol and relevant orthodontic parameters (treatment goal, software, aligner protocol, and any planned extractions); (4) physical adjunct details (type, device/parameters, dose/intensity, frequency, duration, and adherence when reported) and comparator details; (5) outcome definitions, measurement methods, and follow-up time points; (6) numerical results/effect estimates (with dispersion/precision measures as reported) and adverse events; and (7) funding and conflicts of interest when available.

### Evaluate the risk of bias

The risk of bias was evaluated **per outcome** using Cochrane’s RoB 2 [27]. Two independent reviewers (MRS and MON) performed all RoB 2 assessments, with any disagreements resolved by a third reviewer (MYSA). Cochrane emphasizes that study-level summary grades may mask variation in bias across outcomes [26]. Domain-level judgments are reported in S2 Table.

### Evaluating the strength of evidence

To evaluate the certainty of the evidence regarding the efficacy of PAI in CAT, the Grading of Recommendations Assessment, Development, and Evaluation framework (GRADE) was applied [28]. This system classifies the evidence certainty into one of four levels (high, moderate, low, or very low) based on a structured assessment of factors including risk of bias, inconsistency, indirectness, imprecision, and publication bias. Two independent reviewers (MRS and MON) applied GRADE for each outcome (and comparison, where applicable), with any disagreements resolved by a third reviewer (MYSA).

### Summary measures and approach to synthesis

Owing to substantial clinical and methodological heterogeneity across the included studies, the findings were synthesized narratively. Accordingly, in the absence of meta-analysis, findings were synthesized using a structured narrative approach consistent with SWiM principles, including pre-specified grouping, standardized tabulation, transparent presentation of study-level effects, and integration of risk-of-bias and certainty assessments. Outcomes were grouped according to intervention type, comparison, and follow-up period, and similarities and differences in study design, participant characteristics, and outcome definitions were described in a structured way. Extracted data used for the narrative synthesis were obtained using the standardized, pilot-tested extraction form described in the “Study selection and data extraction” subsection; verification and discrepancy resolution procedures are reported there.

## Results

### The search flow and the retrieved studies

**Fig 1:**
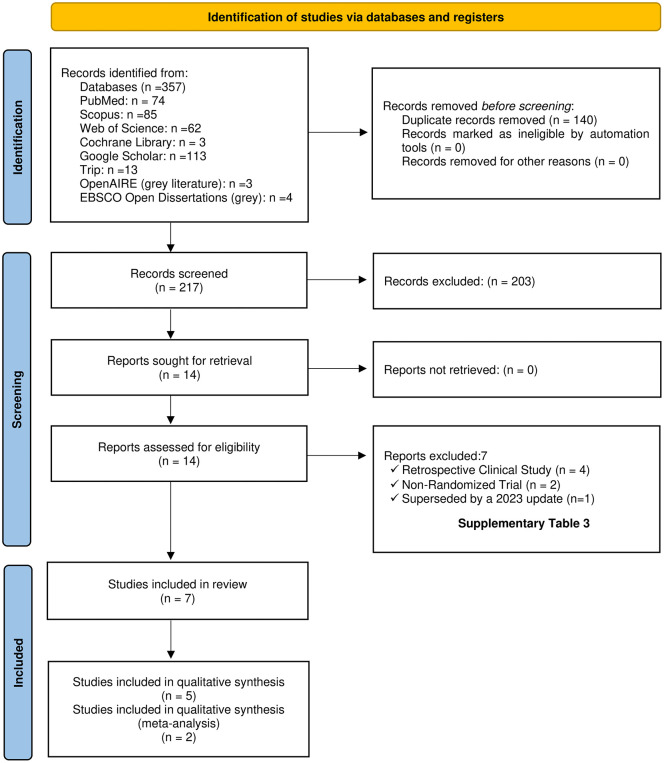
Preferred reporting items for systematic reviews and meta-analyses (PRISMA) flow diagram of the included RCTs. Source: Created by the authors.

The electronic search yielded 357 records. After deduplication, 217 records proceeded to title and abstract screening. Of these, 203 did not meet the eligibility criteria, leaving 14 records for full-text assessment. After a detailed appraisal, seven studies were excluded (reasons in S3 Table); the remaining seven met the inclusion criteria and were retained for this review. The PRISMA flow diagram in Fig 1 summarizes the selection process.

### Characteristics of the included studies

Seven RCTs with 266 participants were included. Interventions covered two modalities: vibration devices such as AcceleDent [19,21,22,29,30] and VPro5 [20], and low-level laser therapy [31]. All trials addressed the leveling and alignment stage of CAT. Outcomes were grouped into six tracks: **1.** objective tooth-movement metrics, including accuracy, efficiency, Little’s Irregularity Index, proximal contact point discrepancy index (PCPDI), and movement-specific measures; **2.** process performance covering completion, tracking, and treatment time; **3.** compliance with device use; **4.** patient-reported outcomes on pain, comfort, and quality of life; **5.** biomarkers from gingival crevicular fluid, including RANKL and OPG; and **6.** periodontal indices reported as plaque index (PI), gingival index (GI), and bleeding on probing (BOP). Characteristics of the included studies are summarized in **Table 2**.

**Table 2:** Characteristics of included RCTs in the systematic review.

Study setting			Orthodontic parameters				Physiologically accelerated intervention specifics				Outcomes studied		Results
Author/ Country/ Design	Treatment comparison	Sample size N (F/M) Ages (years); mean	Treatment Goal	Software	Aligner protocol	Extractions (yes/no)	Intervention device specifications	Follow-up	Duration	Outcome type	Measurement method		
**Alansari S. et al., 2018** **USA** **Parallel**	5 arms: G1: 14-day (control) G2: 7-day sham G3: 7-day + vibration G4: 5-day sham (terminated) G5: 5-day + vibration	Planned n = 75 (15/arm); analyzed ≈ 60 after exclusions; 18–45 y	Alignment Single mandibular incisor planned for labial movement at 0.25 mm per aligner.	Invisalign (SmartTrack); planning ClinCheck; scanning iTero	0.25 mm anteroposterior movement of one mandibular incisor per aligner; four aligners over the study period.	Non-extraction	Device: VPro5 (Propel) 120 Hz, 0.03 g Usage: 5 min/day; sham groups bit on the device inactive	Baseline & end-of-study scans; GCF at end of aligner #2; Pain on days 1 & 3 after each change	20-56 days Until the end of alignment	Tracking (%) vs ClinCheck		Superimpose iTero vs ClinCheck (best-fit), linear deviation on target tooth;	Tracking: Vibration improved tracking vs 7-day no-vibration; 5-day no-vibration arm was halted for poor tracking/discomfort; 5-day with vibration ≈ control. Pain: Early reduction with vibration (days 1 & 3) vs 7-day no-vibration; no other meaningful differences. GCF biomarkers: Broader increases with vibration; higher than control and 7-day no-vibration; no difference between 5- and 7-day vibration.
										Pain		Numeric Rating Scale for pain (0–10);	
										Inflammatory / bone-remodeling markers in GCF		Luminex for GCF	
**Bragassa, 2018** **USA** **parallel**	3 arms G1: 14-day wear (control) G2: 4-day wear G3: 4-day wear + AcceleDent Aura®; aligners ≥22 h/day.	n = 33 (G1 = 10, G2 = 12, G3 = 11); 63 arches analyzed; 64% female; mean age 32.3 ± 9.21 (18–57).	Alignment	Trios 3Shape scanning, ClinCheck, and Geomagic DesignX64 for measurements.	G1: 14 days/aligner. G2–G3: 4 days/aligner. Delivery schedule: T4d (2–4), T2w (5–11), T6w (12–21); wear ≥22 h/day.	Non-extraction	Device: AcceleDent Aura 30 Hz, 0.25 N, 20 min/day.	T0, T4-days, T2-weeks, T6-weeks, T12-weeks.	3 months	% PCPDI reduction (efficiency),		STL models (max/mand) for PCPDI with stated formulas;	Efficiency (PCPDI): accelerated schedule more efficient than conventional; vibration added no clear benefit. PCPDI accuracy: lower with acceleration vs conventional; no meaningful effect of vibration. Overbite (OB) accuracy: no overall differences among arms; simple corrections more accurate than complex. Pain/analgesics: only transient, limited differences; vibration did not reliably reduce pain. Compliance: device use declined over time; aligner wear similar across groups.
										% Accuracy of PCPDI reduction,			
										Pain/analgesics;		Faces Pain Scale® / VAS	
										Accuracy of OB correction.		STL models (pre/post) + ClinCheck; palatal-rugae superimposition; OB measured incisal-to-incisal; %accuracy = achieved OB change / planned OB change.	
										Compliance		Device compliance via FastTrac®.	
**Caccianiga et al. 2016** **Italy** **parallel**	2 arms G1: LLLT + 12h aligners G2: 12h aligners alone	21 (12F/9M) 17–41 years (Mean 26 ± 5.4)	Alignment 6 mm or less of crowding	Clin Check with “3Shape” software	12 hrs/day	Non-extraction	Device: Diode laser LLLT Specs: 980 nm wavelength, 1W power, continuous wave Energy Density: 150 J/cm² (per dental arch) Exposure Duration: 150 seconds (per dental arch) Usage: Biweekly	Biweekly	Until the end of alignment	Number of aligners fitted correctly per patient		• Clinical examination every 2 weeks by the same orthodontist. • Criteria: Passive fit and full tooth coverage.	Fit/success: PBM with 12-h wear maintained fit and enabled series completion; control failed early on 12-h wear. Progression/aligner count: greater progression with PBM; control discontinued. Within-protocol duration: completed with PBM; not completed without PBM at 12-h wear.
										Treatment duration (weeks)		Total weeks from treatment start until: -Laser group: Completion of all aligners (22.1 ± 1). - Control group: Failure point (aligner 3.6 ± 0.8).	
**Idarraga A. et al., 2023** **Spain** **Parallel**	3 arms G1: Vibration from onset + 7-day changes for 6 wks → then 14-day to week 18. G2: Vibration 12 wks from onset; 14-day first 6 wks → 7-day next 6 wks → 14-day to week 18. G3: No vibration; 7-day first 12 wks → 14-day to week 18. Wear ≥22 h/day.	n = 45: G1 = 14, G2 = 15, G3 = 16; female 57.7%; 21-50 years, mean age 30.82 ± 8.33 y	Alignment single mandibular incisor planned for labial movement at 0.25 mm per aligner.	Invisalign®; ClinCheck® planned at 0.25 mm/aligner for the moving lower incisor.	Per-arm 7- vs 14-day schedules as above; 18-week study; wear ≥22 h/day.	Not mentioned	Device: AcceleDent Aura 30 Hz, 0.3 N (≈25 g Usage: 20 min/day (A & B only), per regimen.	T0 baseline, T1 4 wks, T2 6 wks, T3 12 wks, T4 18 wks; (Arm A: no T3 sample).	18 weeks	RANKL and OPG concentrations in GCF.		GCF from a moving lower incisor at ClinCheck-defined pressure point: paper tip 1 mm for 30 s; frozen, ELISAs (MyBioSource® RANKL; Elabscience® OPG) with uniform dilution; concentrations in µg/ml.	RANKL/OPG: no significant differences between arms or over time; nonsignificant trend of ↑RANKL and ↓OPG with vibration (stronger at onset). 7-day vs 14-day schedule: no meaningful differences in biomarkers. Clinical indices (Plaque/Gingival/BOP): no significant within- or between-group changes.
										Gingival/plaque indices and BOP.		Clinically on the same teeth (the target tooth and the two adjacent teeth).	
**Katchooi M. et al., 2018 USA/Canada** **Parallel**	2 arms G1: Active AcceleDent Aura (30 Hz, 0.25 N) G2: Sham (audible motor, zero amplitude) with Invisalign on a weekly change regimen.	n = 27; analyzed 26 (13/13); (18 years or older, mean age ≈ 33 y; balanced sex; ≤ 25 initial aligners.	Alignment	Invisalign® / ClinCheck®; movement per aligner ≤ 0.25 mm; iTero scans pre/post initial series.	7-day changes; 3 aligners per visit, 3-weekly fit checks with predefined lack-of-fit criteria; failures switched to 14-day (off-trial).	Not mentioned	Device: AcceleDent Aura active 30 Hz, ~ 0.25 N Usage: 20 min/day device; aligner wear ≈ 20–21 h/day (questionnaires/Blue-dot indicator).	Every 3 weeks for fit + 3 new aligners; Pain daily for 7 days at baseline and midpoint sets; QoL at baseline/midpoint/end; digital scans pre/post series.	Until the end of treatment	% completion of the weekly regimen.		share completing the 7-day regimen without slowdowns, verified at 3-weekly fit checks.	Weekly completion: no difference between active vibration and sham. Final alignment / change: no meaningful differences. Pain: only minor, transient differences; limited clinical impact. Oral-health QoL: no substantive differences. Compliance (wear/device logs): comparable between groups.
										Little’s Irregularity Index (Mx/Md)		Digital models in OrthoCAD (Little’s II) pre/post;	
										Pain (NRS)		NRS daily ×7 in two periods;	
										QoL		modified OHIP-14	
										Compliance (wear hours/device logs).		device logs + Blue-dot + self-report.	
**Lombardo L. et al., 2018** **Italy** **Parallel**	3 arms G1: 14-day changes (control) G2: 14-day + 20 min/day LFV G3: 7-day + 20 min/day LFV; wear 22 h/day.	n = 45 (15/arm); age 27.1 ± 9.0 y; 20 M/25 F; no dropouts.	Alignment	Trios scanning; setup OrthoAnalyzer (3Shape); F22 aligners; attachments/IPR (≤2 mm/arch); analysis on VAM models.	Staging per aligner: 2° rotation, 2.5° VL/MD tip, 0.2 mm linear; monthly check-ups to series completion.	Non-extraction	Device: AcceleDent 30 Hz, 0.25 N; 20 min/day Usage: Logged compliance: 13.7 min/day (B) and 15.2 min/day (C) on average.	Pre- and post-treatment models only; monthly visits; no intermediate accuracy time-points.	Until end of treatment	Accuracy / imprecision of MD tip / VL tip / rotation by tooth type (both arches).		Mark 100 landmarks; compute MD/VL tip & rotation; derive Prescription & Imprecision; Accuracy = 1 − (Imprecision/Prescription); exclude moves <2°; reliability via Dahlberg & paired t-test.	Maxilla: 7-day + vibration ≈ 14-day without vibration; 14-day + vibration improved accuracy for selected movements (incisor rotation; canine/molar tipping). Mandible: no clear differences among arms. Safety/Compliance: no adverse events; device use was monitored at visits.
**Pescheret, 2017** **USA** **parallel**	2 arms G1: Invisalign + AcceleDent G2: Invisalign alone	Started 40; analyzed 36 (18 vs 18); F = 22 (12/10), M = 14 (6/8). 13-50 years, 22.35	Alignment 6 mm or less of crowding	ClinCheck® + OrthoCAD	Experimental: 7-day changes (10 days if non-compliant); Control: 14-day changes.	Non-extraction	Device: AcceleDent Aura 0.25 N at 30 Hz; Usage: baseline; T2 ≈ 3 mo; T3 ≈ 6 mo; T4 at 12 mo or end	baseline; T2 ≈ 3 mo; T3 ≈ 6 mo; T4 at 12 mo or end	Until end of alignment 12 months	Little’s Irregularity		via OrthoCAD on iTero scans	Alignment: no clear improvement with the device. Pain: gradual mid-term reduction with the device. Time: shorter overall treatment duration with the device. Compliance: variable; device logging not fully reliable.
										pain		via SurveyMonkey	
										Total treatment time		Months from T1 to T2/T3/T4 using iTero scan dates/clinic records.	
										compliance		FastTrac® USB usage report: minutes/day (20 min/day reference) checked at visits.	

AP: anteroposterior; BOP: bleeding on probing; ELISA: enzyme-linked immunosorbent assay; GCF: gingival crevicular fluid; IPR: interproximal reduction; LFV: low-frequency vibration; LLLT: low-level laser therapy; MD: mesio-distal tipping; Mx/Md: maxilla/mandible; NRS: numeric rating scale; OB: overbite; OHIP-14: Oral Health Impact Profile-14; OPG: osteoprotegerin; PCPDI: percent contact-point displacement index; QoL: quality of life; RANKL: receptor activator of nuclear factor-κB ligand; STL: stereolithography model/file; T0: baseline time point; T12w: twelve-weeks time point; T2w: two-weeks time point; T4d: four-days time point; T6w: six-weeks time point; VAM: 3D model analysis software; VAS: visual analogue scale; VL: vestibulo-lingual tipping.

Substantial heterogeneity was observed across included trials. Clinical heterogeneity was present in adjunct modality (vibration vs photobiomodulation/LLLT) and dosing parameters (device settings and daily exposure), as well as aligner-change protocols and treatment schedules. Methodological heterogeneity included study design (parallel-group vs split-mouth), variability in follow-up windows, and non-uniform outcome definitions and measurement platforms (e.g., different tooth-movement metrics, patient-reported scales, and biomarker assays). This heterogeneity limited direct pooling and supported a structured narrative synthesis.

### Risk of bias in the included studies

**Fig 2:**
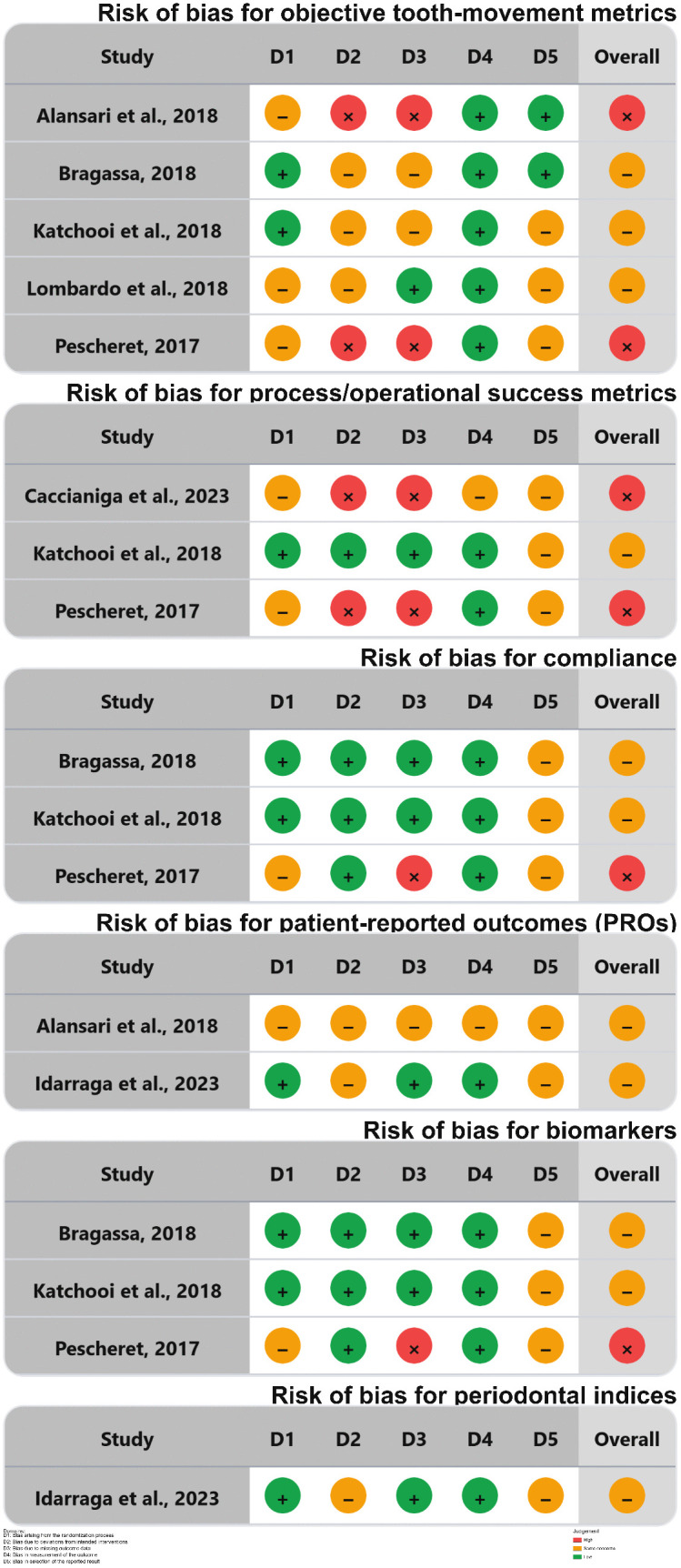
Risk of bias summary of RCTs: the review authors’ judgments about each item of the risk of bias for the included studies using the RoB2 tool. Source: Created by the authors using the RoB-Var tool (MRS Edition).

**Fig 3:**
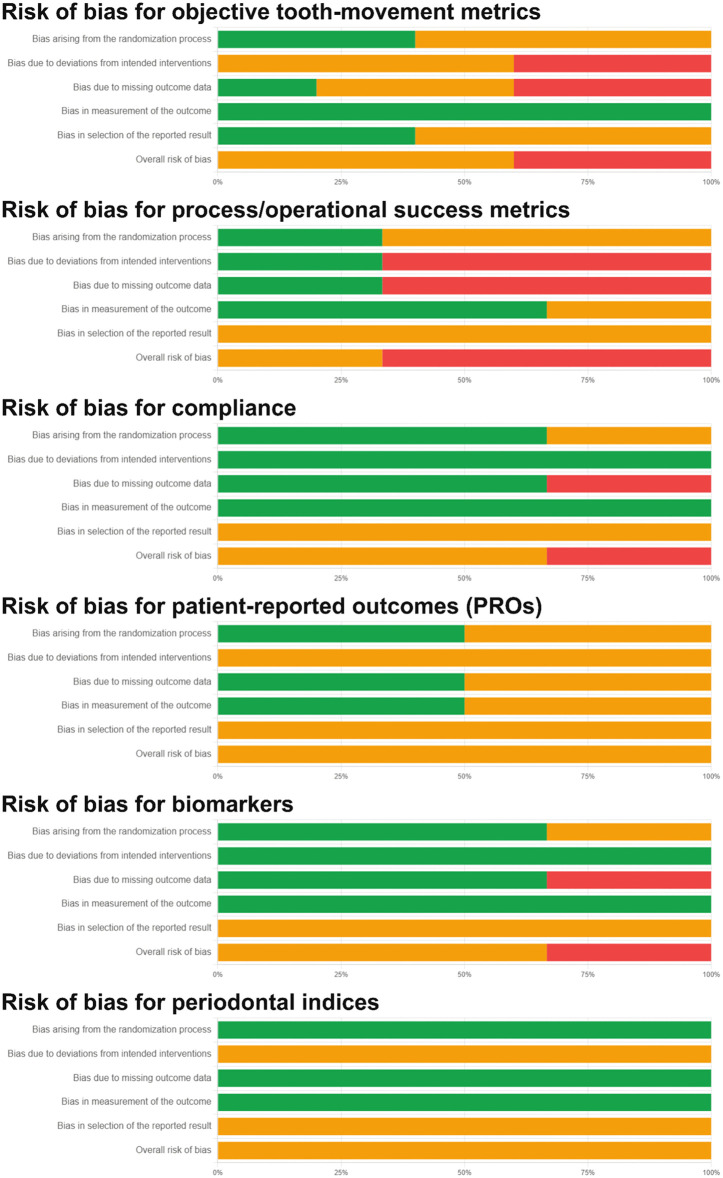
The overall risk of bias score for each field of RCTs: the review authors’ judgments about each item of the risk of bias, presented as percentages across all the studies included. Source: Created by the authors using the RoB-Var tool (MRS Edition).

RoB 2 was applied at the **outcome level** across six tracks. On average, 18 outcomes were assessed, with 10 judged as “some concerns” and 8 as “high risk”. RoB 2.0 assessments are presented in Figs 2–3 (created via RoB-Var tool (MRS Edition) [32]), with detailed rationales provided in S4 Table.

The main sources of bias were concentrated in the trials by Alansari et al. (6), Pescheret et al. (5), and Caccianiga et al. [31], reflecting sensitive issues in domains 2, 3, 4, and 5. Across all tracks, domains 1 and 5 accounted for most “some concerns.” High-risk judgments clustered in domains 2 and 3 when adherence faltered, protocol deviations occurred, or follow-up was incomplete. Domain 4 tended to show lower risk when standardized methods or blinded assessment were used and higher risk when outcomes were patient-reported or adherence-sensitive.

### Main findings of the effect of PAIs on RR

The principal outcomes for PAIs with clear aligners are organized into six tracks (**Table 3**).

**Table 3:** Effects of PAIs on outcomes of CAT (Pooled RCT Data).

Intervention				PAI device	Treatment effect	95% CI	No. of primary trials	No. of events	p value
**Objective tooth-movement metrics**	Little’s Irregularity Index		upper	AcceleDent	0.08	−0.86, 1.02	2 RCTs Katchooi + Pescheret	49	0.869
			Lower		0.44	−0.80, 1.69	2 RCTs Katchooi + Pescheret	48	0.487
	% PCPDI reduction (efficiency)				4d vs 14d: ↑ Efficiency 4d + Vib vs 14d: ↑ Efficiency 4d + Vib vs 4d: Efficiency ≈ no difference	--------------	1 RCT Bragassa	33	0.125 **0.003** 0.089
	% Accuracy of PCPDI reduction				4d vs 14d: ↓ Accuracy 4d + Vib vs 14d: ↓ Accuracy 4d + Vib vs 4d: Efficiency ≈ no difference	---------------	1 RCT Bragassa	33	**0.023** 0.047 0.284
	Accuracy of OB correction				no difference	---------------	1 RCT Bragassa	33	> 0.05
	Accuracy of individual tooth movements (MD/VL tipping & rotation)				**14d + LFV**: selective upper-arch gains: incisor rotation canine VL/MD tip molar VL tip no G1 vs G3 differences lower-arch differences	---------------	1 RCT Lombardo	45	**0.016** **0.007/0.029** **0.0001** > 0.05 > 0.05
	Tracking (%) vs ClinCheck			VPro5 (Propel)	7d Sham ↓ vs control 7d+Vibration ↑ vs 7d Sham 7d+Vibration ≈ control 5d Sham discontinued and lower than control 5d+Vibration ↑ vs 7d Sham 5d+Vibration ≈ control	---------------	1 RCT Alansari	60	**0.022** **0.003** NS **0.022** **0.022** NS
**Process/operational success metrics**	% completion of the weekly regimen.			AcceleDent	Completion high in both arms. No difference between Active and Sham	--------------	1 RCT Katchooi	26	0.999
	Total treatment time (Treatment Time 0–12 mon)				1.562 ± 0.565	0.399, 2.725	1RCT Pescheret	28	**0.010**
	Treatment duration with the 12-hour protocol (weeks)			LLLT	LLLT group 40 ± 2 weeks Control group 7.2 ± 1.6 weeks	--------------	1RCT Caccianiga_2023_	21	Descriptive
	Number of aligners fitted correctly per patient				LLLT group 22.1 ± 1 Control group 3.6 ± 0.8	--------------	1RCT Caccianiga_2023_	21	**0.001**
**Compliance**	Minutes per week			AcceleDent	Active ≈ 121 min/wk vs Sham ≈ 114 min/wk	--------------	1 RCT Katchooi	26	0.390
	Percentage of compliance				Mean ≈ 77% (≈ 15.4 min/day), range 12–121% 54.5% Good Compliers, 45.4% Bad Compliers	--------------	1RCT Pescheret 1 RCT Bragassa	36 33	Descriptive
**Patient-reported outcomes (PROs)**	Pain	NRS	1st day	VPro5 (Propel)	7d+Vibration ↓ pain vs 7d Sham 7d+Vibration ↓ pain vs 14d control other contrasts NS	--------------	1 RCT Alansari	60	**< 0.020** **< 0.034**
			3rd day		7d+Vibration ↓ vs 7d Sham other contrasts NS	--------------	1 RCT Alansari	60	**< 0.026**
			Baseline questionnaire (7 days)	AcceleDent	Only Day-3 difference favoring Active (2.3 ± 1.2 vs 4.2 ± 2.4) other days NS; 7-day sum: 15.3 ± 9.2 vs 21.6 ± 14.1; P = 0.27.	--------------	1 RCT Katchooi	26	**0.026** > 0.05 0.27
			Midpoint questionnaire (7 days)		No significant differences across days Weekly sum: 10.8 ± 11.4 vs 13.0 ± 13.1	--------------	1 RCT Katchooi	26	> 0.05 0.510
		VAS	T1_4 days		No significant reduction with vibration (P > 0.05). Higher in G2 at T2 weeks (P = 0.033). Overall low (< 2.5/10), decreased over time (P = 0.005).	--------------	1 RCT Bragassa	33	> 0.05
			T2_2 weeks						
			T3_ 6 weeks						
			Tfinal_12 weeks						
		Survey Monkey	Average Pain 0–3 mon		0.106 ± 0.287	0.287, −0.478	1RCT Pescheret	35	0.716
			Average Pain 3–6 mon		0.394 ± 0.333	−0.298, 1.087	1RCT Pescheret	35	0.250
			Average Pain 6–12 mon		0.979 ± 0.446	0.013, 1.946	1RCT Pescheret	29	0.047
	Analgesic use				No statistically significant difference between the groups Analgesic medication consumption was the highest at T1 for all groups (G1-14%, G2- 17%, G3 27%).		1 RCT Bragassa	33	0.612
	Oral health–related quality of life (OHIP-14)				No differences between-groups (Active vs Sham) across assessments		1 RCT Katchooi	26	> 0.05
**Biomarkers**	GCF		MILLIPLEX/Luminex	VPro5 (Propel)	End of 2nd aligner: ↑ inflammatory/osteoclastogenic markers in non-vibration arms for some analytes, and larger ↑ with vibration (7d + Vib, 5d + Vib) vs baseline and vs control/sham		1 RCT Alansari	60	**< 0.05**
	OPG		ELISA	AcceleDent	Between-arm: no differences at any time point	--------------	1 RCT Idarraga	45	> 0.05
	RANKL				Between-arm: no differences at any time point		1 RCT Idarraga	45	> 0.05
**Periodontal indices**	PI & GI (ordinal grades) BOP (present/absent)			AcceleDent	No significant within- or between-arm differences;	--------------	1 RCT Idarraga	45	> 0.05

4d: 4-day change schedule; 5d: 5-day change schedule; 7d: 7-day change schedule; 14d: 14-day change schedule; BOP: bleeding on probing; CAT: clear alignment treatment; CI: confidence interval; ClinCheck: digital orthodontic treatment plan; ELISA: enzyme-linked immunosorbent assay; G1: group 1; G2: group 2; G3: group 3; GCF: gingival crevicular fluid; GI: Gingival Index; LFV: low-frequency vibration; MD: mesiodistal; NRS: numeric rating scale; NS: not significant; OB: overbite; PCPDI: Proximal Contact Point Discrepancy Index; OPG: osteoprotegerin; PAIs: physical adjunctive interventions; PI: Plaque Index; RANKL: receptor activator of nuclear factor κB ligand; RCT: randomized controlled trial; VAS: visual analogue scale; VL: vestibulolingual. Bold P values indicate nominal statistical significance (p < 0.05).

### Objective tooth-movement metrics (GRADE: very low)

For objective tooth-movement metrics, two RCTs [19,22] found no AcceleDent effect on Little’s Irregularity Index; effect estimates were 0.08 for the maxilla (p = 0.869) and 0.44 for the mandible (p = 0.487).

In Bragassa et al. [21], 4-day brace adjustments versus a 14-day schedule increased efficiency: PCPDI reduction rose from 18.9% to 23.8% and to 29.1% with vibration (p = 0.003). But accuracy fell, with incisor alignment dropping from 48% to 36% and 37% with vibration (p = 0.023 and p = 0.047). Overbite accuracy showed no protocol differences and was better in simpler cases (p > 0.05).

In Lombardo et al. [30], a 7-day change protocol with vibration performed similarly to a 14-day schedule overall, whereas adding vibration to the 14-day protocol improved accuracy for selected upper-arch movements, specifically incisor rotation and canine/molar tipping. In Alansari et al. [20], tracking accuracy with vibration on a 7-day schedule exceeded sham (p = 0.003) and matched the 14-day control, while the 7-day sham arm underperformed the control (p = 0.022).

### Process/operational success metrics (GRADE: very low)

In Katchooi et al. [22], under a 1-week change schedule, the percentage completing the initial aligner series did not differ between active vibration and sham (Fisher’s exact test, p = 0.999), with fit criteria assessed every 3 weeks. In contrast, in Pescheret et al. [19], total treatment time was shorter with vibration, with significant differences over the 0–3, 0–6, and 0–12-month windows (p = 0.004, 0.028, and 0.010, respectively). Caccianiga et al [31] showed that with a 12-h/day wear protocol + photobiomodulation (LLLT), treatment duration averaged 40 ± 2 weeks versus discontinuation of the 12-h protocol in controls at 7.2 ± 1.6 weeks, with markedly better aligner fit (22.1 ± 1.0 vs 3.6 ± 0.8 correctly fitted aligners per patient; p = 0.001).

### Compliance (GRADE: very low)

Regarding compliance, device logs in Katchooi et al showed similar weekly use between the active-vibration and sham arms with no statistically significant difference (p = 0.39) [22]. In Bragassa [21], device use waned across 12 weeks; only 54% met the ≥ 75% adherence threshold. In Pescheret [19], mean compliance was approximately 77% with a wide range (12–121%); however, an internal FastTrac logging error prevented linking usage to specific time points, rendering these figures descriptive and subject to methodological uncertainty.

### Patient-reported outcomes (PROs) (GRADE: very low)

For PROs, Alansari et al. reported lower pain on days 1 and 3 with vibration under a 7-day schedule versus sham (p < 0.020 and p < 0.034, respectively), while other time points were non-significant [20]. Katchooi et al., using a daily numeric rating scale (NRS: 0–10), detected only a small day-3 difference during the baseline week (p = 0.026) with no subsequent significant differences [22]. Bragassa et al. measured pain with a visual analogue scale (VAS) at 4 days and at 2, 6, and 12 weeks, finding no reliable analgesic effect of vibration (p > 0.05) [21]. Pescheret et al., using a VAS + Faces protocol (daily for 3 days, then weekly), observed a general gradual decline in pain over time without durable clinical superiority of the device [19].

Regarding analgesic intake, Bragassa et al observed a peak at the first visit (G1 = 14%, G2 = 17%, G3 = 27%) followed by a decline, with no between-arm differences (p > 0.05) [21], whereas Katchooi et al found no meaningful change in oral-health–related quality of life (OHIP-14) between vibration and sham across follow-up (p > 0.05) [22]. Overall, these findings do not support a consistent or durable analgesic benefit of vibration across studies.

### Biomarkers (GRADE: very low)

Regarding biomarkers, Alansari et al. [20] sampled gingival crevicular fluid (GCF) at baseline and at the end of the second aligner; a multiplex panel showed significant increases from baseline that were greater with vibration (p < 0.05). In contrast, Idarraga et al. [29] measured the RANKL/OPG ratio at T0, 4, 6, 12, and 18 weeks, finding no significant intra- or inter-group differences at any time point (p > 0.05).

### Periodontal indices (GRADE: low)

In Idarraga et al [29], periodontal indices were recorded clinically on the target tooth and the two adjacent teeth and included PI, GI, and BOP. Analyses showed no statistically significant within-group or between-group differences with vibration or without vibration and no differences between the 7-day and 14-day schedules (p > 0.05).

### Quality of evidence across studies

By GRADE (**Table 4**), overall certainty was low to very low, with downgrades for risk of bias (frequent RoB 2 “some concerns”/ “high”), inconsistency (dispersed effect estimates), indirectness (short follow-up and proxy—rather than patient-important—outcomes), and imprecision (few, small RCTs).

**Table 4:** GRADE summary of findings for RCTs of PAIs on outcomes of CAT.

Outcome domain	No. of studies	Risk of bias	Inconsistency	Indirectness	Imprecision	No. of patients	Summary of effect	Overall certainty
**Objective tooth-movement metrics**	5 RCTs	**Very Serious (−2)**	**Serious (−1)**	Not serious (0)	**Serious (−1)**	187	No consistent overall benefit; at most, limited gains in specific upper-arch movements in one trial; most estimates near “no effect.”	Very low^a^
**Process / operational success**	3 RCTs	**Very Serious (−2)**	**Serious (−1)**	**Serious (−1)**	**Serious (−1)**	75	Completion/tracking broadly similar; mixed signals for total time, often under non-standard protocols or surrogate outcomes.	Very low^b^
**Compliance**	3 RCTs	**Very Serious (−2)**	**Serious (−1)**	Not serious (0)	**Serious (−1)**	95	No active–sham difference in one sham-controlled RCT; adherence declines over time; heterogeneous definitions/measurement across trials.	Very low^c^
**Patient-reported outcomes (PROs)**	4 RCTs	**Very Serious (−2)**	Not serious (0)	Not serious (0)	**Serious (−1)**	154	Only small, early differences (day 1–3); no sustained benefit in pain or QoL.	Very low^d^
**Biomarkers** **(GCF panels; RANKL/OPG)**	2 RCTs	**Serious (−1)**	**Serious (−1)**	**Serious (−1)**	**Serious (−1)**	105	Conflicting findings and small, variable effects; surrogate outcomes not directly translating to patient-important benefits.	Very low^e^
**Periodontal indices (PI/GI/BOP)**	1 RCT	Not serious (0)	Not serious (0)	Not serious (0)	**Serious (−1)**	45	No clinically important between-group differences across follow-up.	Low^f^

GRADE: Grading of Recommendations, Assessment, Development and Evaluation; RCTs: Randomized Controlled Trials; PAIs: Physical Adjunctive Interventions; CAT: Clear Aligner Treatment; PROs: Patient-Reported Outcomes; GCF: Gingival Crevicular Fluid; RANKL: Receptor Activator of Nuclear Factor κB Ligand; OPG: Osteoprotegerin; PI: Plaque Index; GI: Gingival Index; BOP: Bleeding on Probing; QoL: Quality of Life.

^a^Decline two levels for risk of bias (some concern in the randomization process [18,19,28], Deviations from Interventions [20,21,28], Missing Outcome Data [20,21], and selection of the reported result [18,19,21,28], with high risk in Deviations from Interventions [18,19], and Missing Outcome Data [18,19]), one level for Inconsistency*, and one level for imprecision***

^b^Decline two levels for risk of bias (some concern in the randomization process [18,29], Measurement of Outcomes [29], and selection of the reported result [18,21,29], with high risk in Deviations from Interventions [18,29], and Missing Outcome Data [18,29]), one level for Inconsistency*, one level for Indirectness**, and one level for imprecision***

^c^Decline two levels for risk of bias (some concern in the randomization process [18], and selection of the reported result [18,20,21], with high risk in Missing Outcome Data [18]), one level for Inconsistency*, and one level for imprecision***

^d^Decline two levels for risk of bias (some concern in the randomization process [18,19], Deviations from Interventions [20], Missing Outcome Data [19], and selection of the reported result [19,21], with high risk in Deviations from Interventions [18,19], Missing Outcome Data [18], Measurement of Outcomes [18–20], and selection of the reported result [18]), and one level for imprecision***

^e^Decline one level for risk of bias (some concern in the randomization process [19], Deviations from Interventions [19,27], Missing Outcome Data [19], Measurement of Outcomes [19], and selection of the reported result [19,27]), one level for Inconsistency*, one level for Indirectness**, and one level for imprecision***

^f^Decline one level for risk of bias (some concern in Deviations from Interventions [27], and selection of the reported result [27]), and one level for imprecision***

* Wide variance of point estimates across studies Study

** Indirectness: Short assessment duration (study time frame), and a difference between desired (Patient' important) and measured outcomes.

*** Imprecision: Limited number of trials and sample size.

## Discussion

This review represents the first systematic synthesis to date that focuses exclusively on PAIs in CAT, restricts the evidence base to RCTs, and appraises risk of bias at the **outcome level**. Taken together, the findings depict a nuanced picture: the overall benefits appear modest, and in many settings, the cadence of the aligner change protocol seems more influential than the adjunct itself. Results vary across outcomes, with inconsistent findings regarding pain and compliance that limit generalizability. The following discussion interprets these patterns across the six predefined tracks, situates them within the broader literature, and clarifies their clinical and research implications.

### Objective tooth-movement metrics

When objective tooth-movement outcomes were considered, differences between adjunctive vibration and control conditions appeared inconsistent and were frequently intertwined with the prescribed aligner-change interval. Shorter change intervals (e.g., 4 days) may increase the number of planned steps per unit time, but they may also increase the likelihood that small tracking discrepancies accumulate, potentially offsetting gains in efficiency [21]. Although vibration may elicit short-term biologic responses, the available trials did not consistently demonstrate additional benefit for whole-arch alignment outcomes or overbite beyond what could be explained by protocol scheduling and adherence [21,22]. Any apparent advantages were restricted to specific movement types and specific scheduling contexts (e.g., upper-arch movements under a 14-day protocol), suggesting that biomechanics and protocol discipline may be stronger determinants of observed differences than the device itself Moreover, end-point alignment indices such as Little’s Irregularity Index may be relatively insensitive to early, transient effects, because mid-course refinements can converge final alignment across study arms [19,22]. Similarly, improvements in tracking were reported mainly under accelerated 7-day schedules, where an adjunct could plausibly support early aligner seating within a shorter adaptation window; however, comparable performance under 14-day intervals without adjuncts is consistent with the possibility that additional biologic adaptation time can achieve similar end results [20]. Overall, these observations should be interpreted cautiously in light of variability in protocols and outcome measurement across studies.

### Process/operational success metrics

Across process/operational endpoints, observed between-arm differences appeared small and were difficult to attribute to the adjunct alone, because several design and implementation factors can strongly influence these metrics. In Katchooi et al. [22], lack of a detectable difference in completion of scheduled weekly aligner changes is plausibly explained by high baseline adherence in both arms (77% vs. 85%; p > 0.999), a participation (awareness) effect, substantial clinician experience with Invisalign, and the possibility that co-interventions (e.g., biting/seating behavior) occurred even in the sham arm; moreover, inclusion of mild-to-moderate cases (≤25 aligners) may have reduced the likelihood of observing adjunct-specific operational gains [22].

In Pescheret [19], the reported reduction in total treatment time appears more consistent with the accelerated 7-day aligner-change protocol than with a device-specific effect. As the study is a Master’s thesis deposited in an institutional repository (grey literature) and the aligner-change schedule is a likely confounder, the estimate is best treated as hypothesis-generating. Future trials should control for change frequency to better isolate the independent effect of PAIs on operational outcomes.

For LLLT, the operational findings of Caccianiga et al. [31] are mechanistically plausible (enhanced remodeling potentially supporting adequate seating under shorter wear schedules), but the single-center design and early control change limit precision; therefore, the observed effect is best viewed as feasibility under optimized conditions rather than a reliable estimate of effect size [31]. Overall, these data suggest that protocol design, case selection, and adherence measurement may be dominant drivers of operational outcomes, and they should be considered explicitly when interpreting adjunct-related effects.

### Compliance

Evidence indicates that possession of an active vibration device does not increase compliance compared with a sham, indicating that usage is driven primarily by patient behavior rather than device mechanics [22]. Compliance tends to decline over time, consistent with the waning novelty effect commonly observed in home-use interventions [21]. Pescheret [19] reported considerable variability and highlighted measurement limitations due to FastTrac logging, underscoring the need for consistent and objective compliance metrics. This aligns with broader orthodontic adherence evidence showing that self-reported wear time can overestimate objectively measured wear time, supporting the use of objective monitoring where feasible [33].

In clinical settings, effective programs combine device use with behavioral supports such as clear goals, reminders, and feedback from usage logs, and they report exposure in standardized units such as minutes per day or the percentage of a 20-minute prescription [34,35]. Behavioral adherence evidence in clear aligner therapy indicates that remote electronic reminders and automated feedback are associated with improved compliance, supporting the role of prompts/cues and feedback in adherence support [36]. Objective monitoring studies further suggest that awareness of wear-time monitoring can itself increase aligner wear time, although wear time may still decline at later visits [37].

For research, improvements in comparability require harmonized definitions of compliance, precise p-value reporting, and the use of mixed-effects modeling to capture longitudinal trends. These strategies are consistent with widely used behavior change techniques (e.g., goal setting, self-monitoring, feedback, and prompts/cues) that have been repeatedly associated with stronger engagement in digital health interventions [38]. Incorporating a run-in period could further mitigate novelty effects and strengthen study validity [39].

### Patient-reported outcomes (PROs)

Across included RCTs, pain-related PROs followed a time-dependent pattern in which discomfort was greatest shortly after an aligner change and then diminished. Any apparent adjunct-associated benefit, when observed, was confined to this early period, with small reductions reported at day 3 under daily monitoring [22] and at days 1/3 under a 7-day schedule with vibration [20].

In contrast, at broader assessment intervals (e.g., 4 days and 2–12 weeks), vibration did not demonstrate a consistent analgesic effect [21], and pain decreased over time regardless of adjunct use when assessed with VAS and Faces scales [19]. Taken together, the available evidence suggests that any pain-related advantage from vibration—if present—may be short-lived and clinically limited, and that pain trajectories are likely influenced by factors inherent to aligner therapy (e.g., change timing, routine analgesic use, minor adjustments, and counseling) rather than by the adjunct alone. The transient and modest nature of these differences is also consistent with the absence of sustained separation in oral health–related quality-of-life outcomes (OHIP-14) [22].

### Biomarkers

Biomarker outcomes were difficult to interpret across the included trials because comparability was limited by differences in sampling timing, analytical platforms, and device dosing, all of which can materially influence measured signals. Early sampling around initial aligner phases may preferentially capture short-lived inflammatory fluctuations, whereas longer follow-up may reflect biologic adaptation with attenuation of detectable between-arm differences; therefore, divergent biomarker patterns across studies may reflect time-window effects as much as (or more than) adjunct-related biology [20,29]. In addition, cross-study interpretation is constrained when biomarker quantification is performed using different assay technologies (e.g., multiplex platforms versus single-analyte ELISA), which can differ in dynamic range and calibration, and may not yield directly interchangeable values across settings [20,29]. Finally, adjunct devices vary substantially in prescribed exposure and mechanical parameters (e.g., frequency and daily duration), which could plausibly produce non-equivalent biologic signatures even when categorized under the same “adjunct” label.

Accordingly, biomarker findings in this evidence base are best treated as supportive, hypothesis-generating signals rather than standalone proof of clinically meaningful benefit—particularly because biomarkers function as surrogate outcomes and require careful linkage to patient-important endpoints.

### Periodontal indices

Periodontal indices provided limited discriminative signal across trial arms, which may be explained largely by measurement and design considerations rather than by a definitive absence of biologic effect. In periodontally healthy adults, baseline periodontal scores are typically low, creating a floor effect in which meaningful improvement is constrained and small fluctuations may fall below detection thresholds [29]. Sensitivity is further reduced when assessments are restricted to the target tooth and adjacent teeth rather than using full-mouth indices, because localized sampling may miss subtle or diffuse changes [29]. In addition, modest sample size and the relatively short observation window for periodontal endpoints can limit precision for detecting small differences. Accordingly, periodontal findings are best interpreted as showing no clear evidence of measurable periodontal change attributable to the tested adjunct/protocols within the study’s measurement constraints, while acknowledging limited sensitivity and imprecision [29]. Improving interpretability in future work would depend on standardized, full-mouth periodontal assessments with adequate follow-up and reporting.

### Limitations

Despite rigorous methodology with comprehensive search and outcome-level risk-of-bias assessment, several limitations persist. The small number of included studies and their limited sample sizes constrain statistical power and generalizability. Outcome-level risk-of-bias assessments revealed concerns in critical domains, particularly challenges in blinding participants to physical interventions and in outcome measurement, which warrant caution when interpreting positive findings. Furthermore, compliance was often measured using subjective reports or unreliable trackers, reducing the reliability of these data. Finally, substantial heterogeneity in intervention protocols and outcome measurements across studies precluded meta-analysis for many endpoints. These issues may attenuate or inflate effect estimates. Priority methodological features to strengthen future trials are outlined below.

Future trials should prioritize methodological features that reduce bias and improve comparability, including:

- prospective registration with a prespecified primary outcome and statistical analysis plan;
- sham-controlled designs with robust allocation concealment and blinded outcome assessment where feasible;
- standardized reporting of adjunct “dose” (device parameters and minutes/day) with objective exposure logging;
- designs that isolate the adjunct effect from aligner-change cadence (e.g., standardized cadence across arms or factorial designs);
- harmonized, clinically relevant outcome definitions and time points (including objective movement metrics and process endpoints such as refinements and total time);
- adequate follow-up duration and transparent handling of missing data (e.g., intention-to-treat with appropriate longitudinal modeling).

## Conclusions

Randomized evidence to date does not demonstrate a consistent, clinically meaningful adjunct-specific benefit of physical adjunctive interventions in clear aligner therapy. Confidence in the estimated effects is limited by outcome-level risk-of-bias concerns and low to very low certainty of evidence. Consequently, the clinical utility of PAIs for improving CAT outcomes remains unconfirmed.

## Supporting information

S1 Table Electronic search strategy. (DOCX)

S2 Table The RoB 2.0 tool domains and judgments. (DOCX)

S3 Table Studies excluded and reasons for exclusion. (DOCX)

S4 Table Risk of bias of the included RCTs in this systematic review, with supporting reasons. (DOCX)

S1 File PRISMA checklist. Completed PRISMA 2020 checklist. (DOCX)

## Abbreviations

AJODO: American Journal of Orthodontics & Dentofacial Orthopedics
BOP: Bleeding on Probing
CAT: Clear Aligner Treatment
CENTRAL: Cochrane Central Register of Controlled Trials
CONSORT: Consolidated Standards of Reporting Trials
EJO: European Journal of Orthodontics
ELISA: Enzyme-Linked Immunosorbent Assay
GCF: Gingival Crevicular Fluid
GRADE: Grading of Recommendations, Assessment, Development and Evaluation
GI: Gingival Index
LIES: Low-Intensity Electrical Stimulation
LII: Little’s Irregularity Index
LLLT: Low-Level Laser Therapy
NRS: Numeric Rating Scale
OHIP-14: Oral Health Impact Profile-14
OPG: Osteoprotegerin
PAIs: Physical Adjunctive Interventions
PBMT: Photobiomodulation Therapy
PDL: Periodontal Ligament
PEMF: Pulsed Electromagnetic Fields
PI: Plaque Index
PRISMA: Preferred Reporting Items for Systematic Reviews and Meta-Analyses
PROSPERO: International Prospective Register of Systematic Reviews
RANKL: Receptor Activator of Nuclear Factor κB Ligand
RCT(s): Randomized Controlled Trial(s)
RevMan: Review Manager (Cochrane software)
RoB 2: Revised Cochrane Risk-of-Bias Tool for Randomized Trials
VAS: Visual Analogue Scale
VPro5: High-frequency vibration device (brand name)
Wnt/β-catenin: Canonical Wnt/β-catenin signaling pathway.
