# Supplementary material for: The impact of physical adjunctive interventions on outcomes of clear aligner treatment: A systematic review of randomized controlled trials
Source: PLoS One. 2026 Apr 8;21(4):e0346566. doi: 10.1371/journal.pone.0346566 (PMC13061203; doi:10.1371/journal.pone.0346566)
Supplement: S1 Table — (DOCX) [file pone.0346566.s001.docx]

| **S1 Table.** Electronic search strategy | | |
| --- | --- | --- |
| **No** | **Source** | **Search strategy (sets for this database)** |
| 1 | **PubMed / MEDLINE**  15-06-2025 | **#1** = ("clear aligner*"[tiab] OR "invisalign"[tiab] OR "esthetic aligner*"[tiab] OR "removable aligner*"[tiab] OR "orthodontic aligner*"[tiab])  **#2** = ("physical intervention*"[tiab] OR "adjunctive therapy"[tiab] OR "non-surgical intervention*"[tiab] OR vibration[tiab] OR photobiomodulation[tiab] OR "low-level laser therapy"[tiab] OR LLLT[tiab] OR "light emitting diode"[tiab] OR LED[tiab] OR "bioelectric stimulation"[tiab] OR "electric stimulation"[tiab] OR "low-intensity pulsed ultrasound"[tiab] OR LIPUS[tiab] OR "pulsed electromagnetic fields"[tiab] OR PEMF[tiab])  **#3** = ("treatment duration"[tiab] OR "treatment time"[tiab] OR "tooth movement rate"[tiab] OR "Little's Irregularity Index"[tiab] OR "IRR"[tiab] OR "PAR index"[tiab] OR pain[tiab] OR "discomfort"[tiab] OR "root resorption"[tiab] OR "periodontal index"[tiab] OR "oral health-related quality of life"[tiab] OR OHRQoL[tiab])  **#4** = ("randomized controlled trial"[pt] OR "randomized controlled trial"[tiab] OR RCT[tiab])  **#5** = **#1 AND #2 AND #3 AND #4** |
| 2 | **Embase**  15-06-2025 | **#1** = ("clear aligner*" OR "invisalign" OR "esthetic aligner*" OR "removable aligner*" OR "orthodontic aligner*"):ti,ab,kw  **#2** = ("physical intervention*" OR "adjunctive therapy" OR "non-surgical intervention*" OR vibration OR photobiomodulation OR "low-level laser therapy" OR LLLT OR "light emitting diode" OR LED OR "bioelectric stimulation" OR "electric stimulation" OR "low-intensity pulsed ultrasound" OR LIPUS OR "pulsed electromagnetic fields" OR PEMF):ti,ab,kw  **#3** = ("treatment duration" OR "treatment time" OR "tooth movement rate" OR "Little's Irregularity Index" OR "IRR" OR "PAR index" OR pain OR "discomfort" OR "root resorption" OR "periodontal index" OR "oral health-related quality of life" OR OHRQoL):ti,ab,kw  **#4** = ("randomized controlled trial" OR RCT):ti,ab,kw  **#5** = **#1 AND #2 AND #3 AND #4** |
| 3 | **Scopus**  15-06-2025 | **#1** = TITLE-ABS-KEY("clear aligner*" OR "invisalign" OR "esthetic aligner*" OR "removable aligner*" OR "orthodontic aligner*")  **#2** = TITLE-ABS-KEY("physical intervention*" OR "adjunctive therapy" OR "non-surgical intervention*" OR vibration OR photobiomodulation OR "low-level laser therapy" OR LLLT OR "light emitting diode" OR LED OR "bioelectric stimulation" OR "electric stimulation" OR "low-intensity pulsed ultrasound" OR LIPUS OR "pulsed electromagnetic fields" OR PEMF)  **#3** = TITLE-ABS-KEY("treatment duration" OR "treatment time" OR "tooth movement rate" OR "Little's Irregularity Index" OR "IRR" OR "PAR index" OR pain OR "discomfort" OR "root resorption" OR "periodontal index" OR "oral health-related quality of life" OR OHRQoL)  **#4** = TITLE-ABS-KEY("randomized controlled trial" OR RCT)  **#5** = **#1 AND #2 AND #3 AND #4** |
| 4 | **Web of Science (Core Collection)**  15-06-2025 | **#1** = TS=( "clear aligner*" OR "invisalign" OR "esthetic aligner*" OR "removable aligner*" OR "orthodontic aligner*" )  **#2** = TS=( "physical intervention*" OR "adjunctive therapy" OR "non-surgical intervention*" OR vibration OR photobiomodulation OR "low-level laser therapy" OR LLLT OR "light emitting diode" OR LED OR "bioelectric stimulation" OR "electric stimulation" OR "low-intensity pulsed ultrasound" OR LIPUS OR "pulsed electromagnetic fields" OR PEMF )  **#3** = TS=( "treatment duration" OR "treatment time" OR "tooth movement rate" OR "Little's Irregularity Index" OR "IRR" OR "PAR index" OR pain OR "discomfort" OR "root resorption" OR "periodontal index" OR "oral health-related quality of life" OR OHRQoL )  **#4** = TS=( "randomized controlled trial" OR RCT )  **#5** = **#1 AND #2 AND #3 AND #4** |
| 5 | **Cochrane CENTRAL**  15-06-2025 | **#1** = ("clear aligner*" OR "invisalign" OR "esthetic aligner*" OR "removable aligner*" OR "orthodontic aligner*")  **#2** = ("physical intervention*" OR "adjunctive therapy" OR "non-surgical intervention*" OR vibration OR photobiomodulation OR "low-level laser therapy" OR LLLT OR "light emitting diode" OR LED OR "bioelectric stimulation" OR "electric stimulation" OR "low-intensity pulsed ultrasound" OR LIPUS OR "pulsed electromagnetic fields" OR PEMF)  **#3** = ("treatment duration" OR "treatment time" OR "tooth movement rate" OR "Little's Irregularity Index" OR "IRR" OR "PAR index" OR pain OR "discomfort" OR "root resorption" OR "periodontal index" OR "oral health-related quality of life" OR OHRQoL)  **#4** = ("randomized controlled trial" OR RCT)  **#5** = **#1 AND #2 AND #3 AND #4** |
| 6 | **Trip Database**  15-06-2025 | ("clear aligner*" OR "invisalign" OR "esthetic aligner*" OR "removable aligner*" OR "orthodontic aligner*") AND ("physical intervention*" OR "adjunctive therapy" OR "non-surgical intervention*" OR vibration OR photobiomodulation OR "low-level laser therapy" OR LLLT OR "light emitting diode" OR LED OR "bioelectric stimulation" OR "electric stimulation" OR "low-intensity pulsed ultrasound" OR LIPUS OR "pulsed electromagnetic fields" OR PEMF) AND ("treatment duration" OR "treatment time" OR "tooth movement rate" OR "Little's Irregularity Index" OR "IRR" OR "PAR index" OR pain OR "discomfort" OR "root resorption" OR "periodontal index" OR "oral health-related quality of life" OR OHRQoL) AND ("randomized controlled trial" OR RCT) |
| 7 | **Google Scholar**  15-06-2025 | ("clear aligner*" OR "invisalign" OR "esthetic aligner*" OR "removable aligner*" OR "orthodontic aligner*") AND ("physical intervention*" OR "adjunctive therapy" OR "non-surgical intervention*" OR vibration OR photobiomodulation OR "low-level laser therapy" OR LLLT OR "light emitting diode" OR LED OR "bioelectric stimulation" OR "electric stimulation" OR "low-intensity pulsed ultrasound" OR LIPUS OR "pulsed electromagnetic fields" OR PEMF) AND ("treatment duration" OR "treatment time" OR "tooth movement rate" OR "Little's Irregularity Index" OR "IRR" OR "PAR index" OR pain OR "discomfort" OR "root resorption" OR "periodontal index" OR "oral health-related quality of life" OR OHRQoL) AND ("randomized controlled trial" OR RCT) |
| 8 | **OpenAIRE (grey literature)**  15-06-2025 | ("clear aligner*" OR "invisalign" OR "esthetic aligner*" OR "removable aligner*" OR "orthodontic aligner*") AND ("physical intervention*" OR "adjunctive therapy" OR "non-surgical intervention*" OR vibration OR photobiomodulation OR "low-level laser therapy" OR LLLT OR "light emitting diode" OR LED OR "bioelectric stimulation" OR "electric stimulation" OR "low-intensity pulsed ultrasound" OR LIPUS OR "pulsed electromagnetic fields" OR PEMF) AND ("treatment duration" OR "treatment time" OR "tooth movement rate" OR "Little's Irregularity Index" OR "IRR" OR "PAR index" OR pain OR "discomfort" OR "root resorption" OR "periodontal index" OR "oral health-related quality of life" OR OHRQoL) AND ("randomized controlled trial" OR RCT) |
| 9 | **EBSCO Open Dissertations (grey)**  15-06-2025 | ("clear aligner*" OR "invisalign" OR "esthetic aligner*" OR "removable aligner*" OR "orthodontic aligner*") AND ("physical intervention*" OR "adjunctive therapy" OR "non-surgical intervention*" OR vibration OR photobiomodulation OR "low-level laser therapy" OR LLLT OR "light emitting diode" OR LED OR "bioelectric stimulation" OR "electric stimulation" OR "low-intensity pulsed ultrasound" OR LIPUS OR "pulsed electromagnetic fields" OR PEMF) AND ("treatment duration" OR "treatment time" OR "tooth movement rate" OR "Little's Irregularity Index" OR "IRR" OR "PAR index" OR pain OR "discomfort" OR "root resorption" OR "periodontal index" OR "oral health-related quality of life" OR OHRQoL) AND ("randomized controlled trial" OR RCT) |
