# Supplementary material for: The impact of physical adjunctive interventions on outcomes of clear aligner treatment: A systematic review of randomized controlled trials
Source: PLoS One. 2026 Apr 8;21(4):e0346566. doi: 10.1371/journal.pone.0346566 (PMC13061203; doi:10.1371/journal.pone.0346566)
Supplement: S2 Table — (DOCX) [file pone.0346566.s002.docx]

| **S2Table.** The RoB 2.0 tool domains and judgments | | |
| --- | --- | --- |
| **Domains** | **Judgments** | |
| **RoB 2.0 tool (For RCTs)** | | |
| 1. Bias arising from the randomization process | **overall** | **Low, Some**  **concerns, High** |
| 1. Bias due to deviations from intended interventions |  |  |
| 1. Bias due to missing outcome data |  |  |
| 1. Bias in measurement of the outcome |  |  |
| 1. Bias in the selection of the reported result |  |  |
| **RoB 2.0 tool: RCTs**: randomized clinical trials; **Low**: if all fields were estimated as "at low risk of bias"; **Some concerns**: if at least one domain was assessed as "some concerns" but not to be at "high risk of bias" for any domain; **High**: if at least one or more fields were estimated as "at high risk of bias" or if there were some concerns for multiple domains in a way that substantially lowered confidence in the result. | | |
