# Supplementary material for: The impact of physical adjunctive interventions on outcomes of clear aligner treatment: A systematic review of randomized controlled trials
Source: PLoS One. 2026 Apr 8;21(4):e0346566. doi: 10.1371/journal.pone.0346566 (PMC13061203; doi:10.1371/journal.pone.0346566)
Supplement: S3 Table — (DOCX) [file pone.0346566.s003.docx]

| **S3 Table.** Studies excluded and reasons for exclusion | | |
| --- | --- | --- |
| **NO** | **Study** | **Reason for exclusion** |
| **1** | Al-Dboush R, Esfahani AN, El-Bialy T; Impact of photobiomodulation and low-intensity pulsed ultrasound adjunctive interventions on orthodontic treatment duration during clear aligner therapy. Angle Orthod 2021;91(5):619-625. doi: 10.2319/112420-956.1. | Retrospective Clinical Study |
| **2** | Bahammam M, El-Bialy T; Effect of Low-Intensity Pulsed Ultrasound (LIPUS) on Alveolar Bone during Maxillary Expansion Using Clear Aligners. Biomed Res Int 2022;2022:4505063. doi: 10.1155/2022/4505063. |  |
| **3** | Kaur H, El-Bialy T; Shortening of Overall Orthodontic Treatment Duration with Low-Intensity Pulsed Ultrasound (LIPUS). J Clin Med 2020;9(5). doi: 10.3390/jcm9051303. |  |
| **4** | Shipley T, Farouk K, El-Bialy T; Effect of high-frequency vibration on orthodontic tooth movement and bone density. J Orthod Sci 2019;8:15. doi: 10.4103/jos.JOS_17_19. |  |
| **5** | Bilello G, Fazio M, Currò G, et al.; The Effects of Low-frequency Vibration on Aligner Treatment Duration: A Clinical Trial. J Int Soc Prev Community Dent 2022;12(3):345-352. doi: 10.4103/jispcd.JISPCD_311_21. | Controlled Clinical Trial |
| **6** | Memè L, Gallusi G, Coli G, et al.; Photobiomodulation to Reduce Orthodontic Treatment Time in Adults: A Historical Prospective Study. Applied Sciences 2022;12:11532. doi: 10.3390/app122211532. |  |
| **7** | Caccianiga G, Crestale C, Cozzani M, et al.; Low-level laser therapy and invisible removal aligners. J Biol Regul Homeost Agents 2016;30(2 Suppl 1):107-13. | Superseded by a 2023 update. |
