# Supplementary material for: The impact of physical adjunctive interventions on outcomes of clear aligner treatment: A systematic review of randomized controlled trials
Source: PLoS One. 2026 Apr 8;21(4):e0346566. doi: 10.1371/journal.pone.0346566 (PMC13061203; doi:10.1371/journal.pone.0346566)
Supplement: S4 Table — (DOCX) [file pone.0346566.s004.docx]

| **S4 Table.** Risk of bias of the included RCTs in this systematic review, with supporting reasons | | | |
| --- | --- | --- | --- |
| **Alansari S. et al., 2018** | | | |
| **Domain \ Outcome** | **Tracking** | **Pain (NRS)** | **GCF biomarkers** |
| **1) Bias arising from the randomization process** | **Some concerns.** Randomized with **block randomization** across five arms, but no details on sequence generation or **allocation concealment** were reported. | **Some concerns.** Same reason. | **Some concerns.** Same reason. |
| **2) Bias due to deviations from intended interventions** | **High risk.** Only data analysts were blinded; participants/clinicians were aware of the assignment. Non-adherent participants were **dismissed** (e.g., <22 h/day wear or missing 1 day of VPro5), and the **5-sham** arm was **stopped** for nontracking/discomfort—consistent with per-protocol handling rather than ITT. | **High risk.** Unblinded participants reporting a subjective outcome + exclusions for (non)adherence. | **Some concerns.** Objective lab measures, but deviations/exclusions could still influence which data were observed/analyzed. |
| **3) Bias due to missing outcome data** | **High risk.** **0/5 completed** in 5-sham; discontinuations linked to **nontracking** and **discomfort**, i.e., plausibly related to outcomes. | **Some concerns.** Discontinuations/non-adherence occurred and may relate to pain, but completeness at days 1–3 is unclear. | **Some concerns.** Baseline and end-of-second-tray sampling reported; completeness per arm not fully detailed. |
| **4) Bias in the measurement of the outcome** | **Low risk.** **Blinded image assessors**; predefined digital workflow; **measurement error < 0.2 mm** with non-significant intra-/inter-observer errors. | **High risk.** **Self-reported pain** with unblinded participants. | **Some concerns.** Objective **MILLIPLEX/Luminex** assays reported; lab blinding not stated. |
| **5) Bias in the selection of the reported result** | **Some concerns.** No public trial registration or pre-specified analysis plan reported in the article; multiple outcomes/analyses. (By RoB 2, absence of a pre-specified plan generally → “some concerns”.) | **Some concerns.** Same reason. | **Some concerns.** Same reason. |
| **Overall RoB** | **High risk** | **High risk** | **Some concerns** |

| **Bragassa, 2018** | | | | | |
| --- | --- | --- | --- | --- | --- |
| **Domain \ Outcome** | **Efficiency of alignment (% PCPDI reduction)** | **Accuracy of alignment (% PCPDI accuracy)** | **Pain/analgesics** | **Overbite (OB) accuracy** | **Compliance (device use)** |
| **1) Bias arising from the randomization process** | **Low**: Independent third-party biostatistician generated the sequence in R with block randomization; allocation concealment by contacting the sequence generator; baseline balance documented. | **Low**: Same randomization and baseline comparability apply to this outcome. | **Low**: Randomization unaffected; lack of blinding is addressed in the measurement domain. | **Low**: The OB part follows the same randomized trial framework and registration. | **Low:** Same. |
| **2) Bias due to deviations from intended interventions** *(effect of assignment)* | **Some concerns**: No sham device → participants/clinicians unblinded; vibration compliance dropped (only 54% “good compliers”), though aligner wear hours were similar across groups. Possible behavior changes cannot be excluded. | **Some concerns**: Same rationale (unblinded participants + variable vibration adherence); ITT-style mixed models used without adherence adjustment. | **Some concerns**: Knowledge of allocation could influence analgesic use/self-report; no sham; analgesics captured, but no measures to prevent behavioural deviations. | **Some concerns**: Same deviation issues (no blinding + adherence) also apply in the OB section. | **Low**: Compliance is a planned process outcome; instructions and counselling were protocolized; analysis reflects what happened. |
| **3) Bias due to missing outcome data** | **Some concerns**: No loss to follow-up, but two participants were excluded post-randomization before analysis because ClinCheck plans did not initiate movement simultaneously; such post-randomization exclusions may introduce limited bias. | **Some concerns**: Same post-randomization exclusions apply to this analysis set. | **Low**: No loss to follow-up; pain/analgesics collected at pre-specified time points for all subjects. | **Some concerns**: In the OB analysis, two cases were excluded after analysis because the predicted OB change over 12 weeks was <0.2 mm (below measurement error), reducing the sample and potentially linked to outcomes. | **Low**: Usage logs available; **compliance fell over time,** but data was captured. |
| **4) Bias in the measurement of the outcome** | **Low**: Objective digital-model outcomes assessed by a blinded investigator on de-identified models. | **Low**: Same blinded, objective measurement process. | **High**: Self-reported pain without participant blinding (no sham) → susceptible to performance/measurement bias. | **Low**: OB measured on digital models with a blinded assessor and stable palatal rugae superimposition. | **Low**: **Objective** FastTrack device logs quantify use. |
| **5) Bias in the selection of the reported result** | **Low**: Trial registered (NCT02868554); outcomes and analyses described a priori. | **Low**: Same (registration + pre-specified methods). | **Low**: Tools and timepoints pre-specified (Faces Pain Scale; analgesic survey at fixed visits). | **Some concerns**: OB analysis labelled “explorative”; “simple vs complex” strata declared before analysis, but multiplicity/subgroup focus raises some concern. | **Some concerns**: “Good complier ≥75%”; **only 54%** met this threshold/summary not pre-registered. |
| **Overall RoB** | **Some concerns** | **Some concerns** | **High risk** | **Some concerns** | **Some concerns** |

| **Caccianiga et al., 2016** | | | | | | | |
| --- | --- | --- | --- | --- | --- | --- | --- |
| **Domain \ Outcome** | **(1) # of correctly fitted aligners** | | **(2) Completion on 12 h/day** | | | **(3) Time on 12 h/day** | |
| **D1: Randomization process** | **Some concerns**: Randomization between laser/control with baseline balance (age/sex/crowding) reported, but **no details** on sequence generation or **allocation concealment**. | | **Some concerns**: Same (insufficient info on sequence/concealment). | | | **Some concerns**: Same randomization description applies here. | |
| **D2: Deviations from intended interventions (effect of assignment)** | **High**: No sham; open-label participants/providers. The control arm was **switched to 22 h/day** to complete treatment, a **major protocol deviation** from the intended 12-h regimen; analysis likely reflects what happened rather than ITT. | | **High**: Same deviation: “completion under 12 h” is **directly impacted** by switching the control to 22 h/day, with unblinded care. | | | **High**: Shorter persistence on 12 h in controls (≈7.2±1.6 weeks) due to early stop/switch; a post-randomization deviation integral to the outcome. | |
| **D3: Missing outcome data** | **High**: After switching, **post-switch data** under 12 h/day are absent/not comparable; missingness is **related to outcome** (poor fit → switch). | | **High**: “Success on 12 h” is censored by early discontinuation in controls; later data under the planned frame are unavailable. | | | **High**: Time-to-stop on 12 h is curtailed in controls for outcome-related reasons (non-fit), i.e., informative missingness. | |
| **D4: Measurement of the outcome** | **Some concerns**: “Fit/passive” judgement made by a clinician **not blinded** to arm; although clinically straightforward, assessor knowledge could bias decisions; no blinded outcome assessment reported. | | **Some concerns**: “Completion” is a clinical decision in an open-label setting; potential measurement/performance bias. | | | **Some concerns**: Weeks-to-stop is time-based (objective) but the **decision to stop** is unblinded and may be influenced by assignment. | |
| **D5: Selection of the reported result** | **Some concerns**: No **trial registration**/**pre-specified analysis plan** described; emphasis on aligner counts/completion without prior specification raises selection concerns. | | **Some concerns**: Same (absence of prereg/SAP). | | | **Some concerns**: Same; duration presented alongside strong causal claims without a documented pre-analysis plan. | |
| **Overall RoB** | **High risk** | | **High risk** | | | **High risk** | |
| **Idarraga A. et al., 2023** | | | | | | | |
| **Domain \ Outcome** | **RANKL (GCF)** | | | **OPG (GCF)** | **Plaque/Gingival/BOP** | | |
| **D1: Bias arising from the randomization process** | **Low**: Computer randomization + **SNOSE** (opaque sealed envelopes) with sequential opening; baseline balance reported. | | | **Low**: Same generation/concealment applies. | **Low** — Same randomized framework covers secondary outcomes. | | |
| **D2: Bias due to deviations from intended interventions (effect of assignment)** | **Some concerns**: Open-label (no sham) and arm-specific **aligner-change schedules**; no explicit adherence analysis reported; however, the outcome is **objective laboratory** data, limiting performance effects. | | | **Some concerns**: Same rationale (unblinded delivery + protocol differences) with objective measurement. | **Some concerns**: Open-label clinical decisions may be influenced by assignment despite assessor blinding. | | |
| **D3: Bias due to missing outcome data** | **Low**: No attrition reported; “NA” at some points (e.g., Group A at T3) is **by design**, not outcome-related missingness. | | | **Low**: Similar completeness per schedule; no outcome-related loss. | **Low**: No differential loss reported across time/arms for these indices. | | |
| **D4: Bias in measurement of the outcome** | **Low**: **Blinded** clinical/lab assessments; duplicate ELISA with stated sensitivities; assessors could not distinguish groups. | | | **Low**: Same blinded laboratory workflow. | **Low**: Secondary indices measured with **blinded assessors** as stated. | | |
| **D5: Bias in the selection of the reported result** | **Some concerns**: Trial **registration (NCT05316636)** and primary outcomes stated, but no public, detailed **pre-specified analysis plan (SAP)**; limited risk of selective analysis remains. | | | **Some concerns**: Same (registration noted; SAP details not public). | **Some concerns**: Secondary outcomes reported without a detailed pre-registration of their analysis. | | |
| **Overall RoB** | **Some concerns** | | | **Some concerns** | **Some concerns** | | |
| **Katchooi M. et al., 2018** | | | | | | | |
| **Domain \ Outcome** | **Weekly-series completion** | **Final alignment / Irregularity change** | | **Pain** | **Quality of life** | | **Compliance (min/week, device logs)** |
| **D1: Randomization process** | **Low**: Block-stratified (age/sex) sequence in **R**; randomization list held off-site; allocation obtained via coordinator → proper concealment; baseline balance acceptable. | **Low**: Same generation/concealment and baseline comparability apply. | | **Low**: Same randomization for pain outcomes. | **Low**: Same randomized framework for QoL. | | **Low**: Same. |
| **D2: Deviations from intended interventions (effect of assignment)** | **Low**: **Participants/providers blinded** by a credible sham; daily 20-min use in both arms, device-logged; no meaningful compliance differences. | **Some concerns**: Outcome assessed **only in completers**; despite good blinding, restricting analysis may reflect unmeasured behavioral deviations before exclusion. | | **Low**: Subjective outcome, but **effective participant blinding (sham)** limits performance/reporting bias; device usage is similar. | **Low**: Triple-blind setting with uniform survey scheduling reduces deviation risk. | | **Low**: **Objective device-logged** use in both arms; identical instructions. |
| **D3: Missing outcome data** | **Low**: No attrition; one exclusion due to device-swap error (not outcome-related). | **Some concerns**: **Completers-only** analysis can induce selection bias if completion relates to outcome; completion rates were similar (77% vs 85%). | | **Low**: Planned diary windows at two stages; no differential missingness affecting arm comparison is reported. | **Low**: Good completeness across time/arms per reported tables. | | **Low**: **All usage logs available**; analyzed by arm and by completer status (Table IV). |
| **D4: Measurement of the outcome** | **Low**: Completion judged by **pre-specified clinical fit criteria** with **blinded assessors** and photographic documentation of misfit. | **Low**: **De-identified digital models** with a **blinded examiner**; Dahlberg error ≈ 0.1 mm. | | **Low**: Although pain is self-reported, **participant blinding** (active vs sham masked) minimizes measurement bias. | **Low**: Adapted OHIP tools administered at fixed times with blinded assessment. | | **Low**: **Objective** FastTrac logs; see figure/table. |
| **D5: Selection of the reported result** | **Some concerns**: Trial registered (**NCT02438280**) but **protocol not published before commencement**; no public detailed SAP. | **Some concerns**: Same reason (registration present; SAP details not public). | | **Some concerns**: Standardized tools/timings, yet absent pre-published SAP leaves limited selection risk. | **Some concerns**: As above (registered, but no publicly detailed pre-analysis plan). | | **Some concerns**: No pre-specified plan for summarizing compliance. |
| **Overall RoB** | **Some concerns** | **Some concerns** | | **Some concerns** | **Some concerns** | | **Some concerns** |
| **Lombardo L. et al., 2018** | | | | | | | |
| **Domain \ Outcome** | **Upper incisor rotation accuracy + Upper canine VL/MD tipping accuracy + Upper molar VL tipping accuracy** | | | | | | |
| **D1: Bias arising from the randomization process** | **Some concerns**: Randomized by an **external statistician** using **Stata,** with 15 participants per arm enforced; **allocation concealment not reported**. Baseline generally balanced, with a **female excess in group C**. | | | | | | |
| **D2: Bias due to deviations from intended interventions (effect of assignment)** | **Some concerns**: **Open-label** for patients/clinicians (no sham). **Sub-target device use** (mean **13.7 and 15.2 min/day** in B and C) and **verbal boosting** at monthly checks could induce behavioral deviations. | | | | | | |
| **D3: Bias due to missing outcome data** | **Low**: **No dropouts** (45/45 completed). Exclusion of movements <2° was a **pre-specified, uniform method-sensitivity threshold**. | | | | | | |
| **D4: Bias in measurement of the outcome** | **Low**: **Blinded** outcome analyst; objective **VAM** digital-model workflow with repeatability checks (Dahlberg), no systematic error. | | | | | | |
| **D5: Bias in the selection of the reported result** | **Some concerns**: **Trial registered** (DRK00015613) but **no publicly detailed pre-specified analysis plan** in the article; multiplicity across many teeth/movements leaves limited selection risk. (RoB 2 guidance notes under-reporting/SAP absence often warrants “some concerns”.) | | | | | | |
| **Overall RoB** | **Some concerns** | | | | | | |
| **Pescheret, 2017** | | | | | | | |
| **Domain \ Outcome** | **Anterior alignment (Little’s Index)** | **Pain** | | | **Total treatment time** | | **Compliance (device use, FastTrac %)** |
| **D1: Bias from the randomization process** | **Some concerns**: Allocation by drawing straws; no formal report of concealment/central sequence. However, the immediate chance draw offers momentary concealment (next assignment not knowable pre-draw), and no baseline imbalances suggesting subversion were evident. | **Some concerns**: Allocation by drawing straws; no formal report of concealment/central sequence. However, the immediate chance draw offers momentary concealment (next assignment not knowable pre-draw), and no baseline imbalances suggesting subversion were evident. | | | **Some concerns**: Allocation by drawing straws; no formal report of concealment/central sequence. However, the immediate chance draw offers momentary concealment (next assignment not knowable pre-draw), and no baseline imbalances suggesting subversion were evident. | | **Some concerns**: Allocation by drawing straws; no formal report of concealment/central sequence. However, the immediate chance draw offers momentary concealment (next assignment not knowable pre-draw), and no baseline imbalances suggesting subversion were evident. |
| **D2: Bias due to deviations from intended interventions (effect of assignment)** | **High**: **Different aligner-change schedules** by arm (7 vs 14 days) and **10-day rescue schedule** for non-adherent wearers; participants unblinded. These post-randomization deviations plausibly affect alignment speed. | **High**: Unblinded self-report + schedule differences likely influence perceived pain. | | | **High**: Between-arm schedule differences (7/14/10 days) confound treatment time as an effect of assignment. | | **Not applicable / note**: Compliance here is descriptive within the experimental arm (not a between-arm effect estimate). These reminders/incentives were protocolized for both groups; thus, **judged Low** for this domain. |
| **D3: Bias due to missing outcome data** | **High**: 4 dropouts (3 experimental, 1 control) with reasons tied to post-randomization adherence; no strategy to address missingness. | **High**: Pain recorded only during the “experimental period,” with dropouts and no handling of missingness. | | | **High**: Appears completers-only; missingness linked to adherence. | | **High**: **Drop rule at <50%** device use: participants could be removed for low compliance, so missingness depends directly on the outcome value. |
| **D4: Bias in measurement of the outcome** | **Low**: De-identified digital models; objective Little’s Index in OrthoCAD. | **High**: Unblinded self-reported pain via SurveyMonkey. | | | **Low**: Time abstracted from records (objective). | | **Low**: Compliance objectively measured via **FastTrac** device logs (daily % downloaded). |
| **D5: Bias in the selection of the reported result** | **Some concerns**: No publicly pre-specified protocol/SAP. | **High**: **Analysis method switched** post-hoc (weekly averages → per-aligner averages) with no pre-specification. | | | **Some concerns**: No pre-specified plan reported. | | **Some concerns**: No pre-specified plan for summarizing compliance; computed mean daily % a posteriori. |
| **Overall RoB** | **High risk** | **High risk** | | | **High risk** | | **High risk** |
