# Supplementary material for: The impact of physical adjunctive interventions on outcomes of clear aligner treatment: A systematic review of randomized controlled trials
Source: PLoS One. 2026 Apr 8;21(4):e0346566. doi: 10.1371/journal.pone.0346566 (PMC13061203; doi:10.1371/journal.pone.0346566)
Supplement: S1 File — (DOCX) [file pone.0346566.s005.docx]

| **Section and Topic** | **Item #** | **Checklist item** | **Location where item is reported** |
| --- | --- | --- | --- |
| **TITLE** | | |  |
| Title | 1 | The Impact of Physical Adjunctive Interventions on Outcomes of Clear Aligner Treatment: A Systematic Review and Meta-Analysis of Randomized Controlled | p. 1 (Title) |
| **ABSTRACT** | | |  |
| Abstract | 2 | Abstract: Background: Physical adjunctive interventions (PAIs)—such as vibration and low-level laser—are promoted to accelerate tooth movement, stabilize tracking, and reduce discomfort in clear aligner treatment (CAT), but randomized evidence is mixed. Objective: To synthesize randomized controlled trials (RCTs) on PAIs used with CAT and appraise risk of bias and certainty of evidence at the outc | p. 2-3 (Abstract) |
| **INTRODUCTION** | | |  |
| Rationale | 3 | Rationale summarized: challenges in CAT efficiency and claims for PAI; gap in randomized evidence. | p. 15-6-7  (BACKGROUND) |
| Objectives | 4 | Objective: To synthesize randomized controlled trials (RCTs) on PAIs used with CAT and appraise risk of bias and certainty of evidence at the outcome level. | p. 2 -7  (Abstract) |
| **METHODS** | | |  |
| Eligibility criteria | 5 | Inclusion: Human randomized controlled trials only, using either parallel-group or split-mouth designs.. Participants: Healthy individuals of any age, sex, or ethnicity undergoing clear aligner therapy, with or without extraction plans; no restrictions on malocclusion type or treatment indication.. Interventions: Nonsurgical physical adjuncts intended to accelerate orthodontic tooth movement (e.g. | p. 8 (Eligibility criteria) |
| Information sources | 6 | Databases/registers: PubMed, Embase, Scopus, Web of Science, CENTRAL, Trip, Google Scholar; last search June 2025; plus grey literature and hand-searching. | p. 9 (Search Strategy) |
| Search strategy | 7 | Full strategies (databases, filters/limits) reported; supplementary table cited. | p. 9 (Search Strategy) |
| Selection process | 8 | Two reviewers screened titles/abstracts and full texts independently; disagreements resolved by discussion/third reviewer. | p. 9 (Study selection and data extraction) |
| Data collection process | 9 | Duplicate extraction with piloted forms; one extracted, second verified; contacted authors if needed; conflicts resolved. | p. 9 (Study selection and data extraction) |
| Data items | 10a | Outcomes: Total treatment time, Little’s Irregularity Index, PCPDI (reduction), tracking accuracy, pain, oral health–related quality of life, biomarkers, periodontal indices, and root resorption. | p. 9 (Eligibility criteria) |
|  | 10b | Other variables: sample size, age/sex, malocclusion, aligner regimen, PAI device/parameters, follow-up, measurement methods. | p. 9 (Study selection and data extraction) |
| Study risk of bias assessment | 11 | Outcome-level RoB 2 applied by two reviewers; domains included randomization, deviations, missing data, measurement, reporting. | p. 9-10 (Risk of Bias) |
| Effect measures | 12 | Effect measures: mean differences with 95% CIs for continuous outcomes; random-effects model in RevMan 5.4.1. | p. 10 (Effect measures) |
| Synthesis methods | 13a | Eligibility for each synthesis pre-specified by outcome/measure; only comparable RCT arms pooled. | p. 10 (Summary measures and approach to synthesis) |
|  | 13b | Data prepared/harmonized; SDs derived from SE/IQR where needed; units aligned. | p. 10 (Summary measures and approach to synthesis) |
|  | 13c | Random-effects meta-analyses; heterogeneity via τ²/I²; planned subgroups/sensitivity; software RevMan 5.4.1. | p. 10 (Summary measures and approach to synthesis) |
|  | 13d | Study selection into each synthesis documented; sensitivity excluding high RoB studies. | p. 10 (Summary measures and approach to synthesis) |
|  | 13e | Explored heterogeneity by device (vibration/LLLT), arch, and aligner cadence (e.g., 7-day vs longer). | p. 10 (Summary measures and approach to synthesis) |
|  | 13f | Sensitivity analyses: exclude high RoB, alternative models, leave-one-out where applicable. | p. 10 (Summary measures and approach to synthesis) |
| Reporting bias assessment | 14 | Reporting bias planned if ≥10 trials per outcome (funnel/Egger); not feasible otherwise. | p. 10 (Summary measures and approach to synthesis) |
| Certainty assessment | 15 | Certainty appraised with GRADE at outcome-level by two reviewers; downgrades for bias, inconsistency, imprecision, reporting bias. | p. 10 (GRADE) |
| **RESULTS** | | |  |
| Study selection | 16a | Records identified: 357; after deduplication: 217; screened: 203; see PRISMA flow. | p. 11 (RESULTS) |
|  | 16b | List of excluded full-texts with reasons in PRISMA flow/appendix (e.g., non-RCTs, wrong intervention/population). | p. 11 (RESULTS) |
| Study characteristics | 17 | Seven RCTs (n≈266); CAT with vibration/LLLT; change cadence mostly 7–10 days; follow-up to completion or predefined movement. | p. 11 (Study characteristics) |
| Risk of bias in studies | 18 | RoB 2 outcomes varied; common issues: allocation concealment, blinding infeasibility, selective reporting. | p. 14 (RESULTS) |
| Results of individual studies | 19 | Per-trial estimates (LII, tracking, pain VAS, completion, compliance, periodontal indices) with 95% CIs tabulated/figured. | p. 14-15 (RESULTS) |
| Results of syntheses | 20a | Pooled effects show no significant improvement in LII; tracking improved with vibration under 7-day schedule only. | p. 15-16-17-18 (RESULTS) |
|  | 20b | Effects small/inconsistent; heterogeneity low–moderate; results broadly robust to sensitivity checks. | p. 15-16-17-18 (RESULTS) |
|  | 20c | Subgroups: device type and cadence; sensitivity excluding high RoB maintained conclusions. | p. 15-16-17-18 (RESULTS) |
|  | 20d | Limitations: small samples, outcome non-uniformity, few trials; limited power for bias detection. | p. 15-16-17-18 (RESULTS) |
| Reporting biases | 21 | Reporting bias not assessed (number of studies per outcome <10). | p. 14 (RESULTS) |
| Certainty of evidence | 22 | Certainty: very low to low across outcomes (downgrades for bias, inconsistency, imprecision). | p. 19 -20 (DISCUSSION) |
| **DISCUSSION** | | |  |
| Discussion | 23a | Interpretation: PAIs do not deliver consistent clinical gains in CAT; cadence remains main driver. | p. 21 (DISCUSSION) |
|  | 23b | Evidence limitations: small/heterogeneous RCTs, short follow-up, inconsistent reporting. | p. 21-22-23-24-25 (DISCUSSION) |
|  | 23c | Review process limits: potential publication/language bias despite broad search. | p. 25-26 (DISCUSSION) |
|  | 23d | Implications: routine PAI use not supported; need preregistered, adequately powered, standardized RCTs. | p. 21-22-23-24-25 (DISCUSSION) |
| **OTHER INFORMATION** | | |  |
| Registration and protocol | 24a | Registration: PROSPERO CRD xxxxxxxxxxx. | p. 2 -7 (Registration) |
|  | 24b | Protocol availability noted in Methods; prospective registration cited. | p. 7 (Materials and methods) |
|  | 24c | Any amendments would be documented in Methods (if applicable). | p. 7 (Materials and methods) |
| Support | 25 | Funding/source of support stated under Funding. | p. 30 (Funding) |
| Competing interests | 26 | Competing interests/ethics statements under Declarations. | p. 30 (Declarations) |
| Availability of data, code and other materials | 27 | Data availability statement provided; data within article/supplements; more on request. | p. 30 (Data availability) |

*From:*  Page MJ, McKenzie JE, Bossuyt PM, Boutron I, Hoffmann TC, Mulrow CD, et al. The PRISMA 2020 statement: an updated guideline for reporting systematic reviews. BMJ 2021;372:n71. doi: 10.1136/bmj.n71
